# Plant Hsp90 is a novel adjuvant that elicits a strong humoral and cellular immune response against B- and T-cell epitopes of a *Toxoplasma gondii* SAG1 peptide

**DOI:** 10.1186/s13071-019-3362-6

**Published:** 2019-03-25

**Authors:** Edwin F. Sánchez-López, Mariana G. Corigliano, Romina M. Albarracín, Valeria A. Sander, Ariel Legarralde, Sofía A. Bengoa-Luoni, Marina Clemente

**Affiliations:** Unidad de Biotecnología 6-UB6, IIB-INTECH, CONICET-UNSAM, Intendente Marino Km 8.2, B7130IWA Chascomús, Buenos Aires Province Argentina

**Keywords:** Plant Hsp90, Adjuvant, Vaccine, Epitopes, SAG1, Toxoplasmosis, *Toxoplasma gondii*

## Abstract

**Background:**

The 90-kDa heat-shock protein (Hsp90) from *Nicotiana benthamiana* (NbHsp90.3) is a promising adjuvant, especially for those vaccines that require a T cell-mediated immune response. *Toxoplasma gondii* SAG1 is considered one of the most important antigens for the development of effective subunit vaccines. Some epitopes located in the SAG1 C-terminus region have showed a strong humoral and cellular immune response. In the present study, we aimed to assess the efficacy of NbHsp90.3 as carrier/adjuvant of SAG1-derived peptide (SAG1_HC_) in a *T. gondii* infection murine model.

**Methods:**

In the present study, C57BL/6 mice were intraperitoneal immunized with the NbHsp90.3-SAG1_HC_ fusion protein (NbHsp90.3-SAG1_HC_ group), mature SAG1 (SAG1m group), NbHsp90.3 (NbHsp90.3 group) or PBS buffer 1× (PBS group). The levels of IgG antibodies and the cytokine profile were determined by ELISA. Two weeks after the last immunization, all mice were orally challenged with 20 cysts of *T. gondii* Me49 strain and the number of brain cysts was determined. In addition, both humoral and cellular immune responses were also evaluated during the acute and chronic phase of *T. gondii* infection by ELISA.

**Results:**

The characterization of the immune response generated after vaccination with NbHsp90.3 as an adjuvant showed that NbHsp90.3-SAG1_HC_-immunized mice produced antibodies that were able to recognize not only rSAG1m but also the native SAG1 present in the total lysate antigen extract (SAG1_TLA_) from *T. gondii* tachyzoites, while control groups did not. Furthermore, anti-rSAG1m IgG2a/2b antibodies were significantly induced. In addition, only the spleen cell cultures from NbHsp90.3-SAG1_HC_-immunized mice showed a significantly increased production of IFN-γ. During the chronic phase of *T. gondii* infection, the antibodies generated by the infection were unable to detect the recombinant protein, but they did react with TLA extract. In addition, splenocytes from all groups showed a high production of IFN-γ when stimulated with rGRA4, but only those from NbHsp90.3-SAG1_HC_ group stimulated with rSAG1m showed high production of IFN-γ. Finally, NbHsp90.3-SAG1_HC_-immunized mice exhibited a significant reduction in the cyst load (56%) against *T. gondii* infection.

**Conclusions:**

We demonstrated that NbHsp90.3 enhances the humoral and cell-mediated immune response through a Th1 type cytokine production. Mice vaccinated with NbHsp90.3-SAG1_HC_ exhibited a partial protection against *T. gondii* infection and it was correlated with the induction of memory immune response. We developed and validated a vaccine formulation which, to our knowledge, for the first time includes the NbHsp90.3 protein covalently fused to a peptide from *T. gondii* SAG1 protein that contains T- and B-cell epitopes.

**Electronic supplementary material:**

The online version of this article (10.1186/s13071-019-3362-6) contains supplementary material, which is available to authorized users.

## Background

SAG1 is present in tachyzoites of *T. gondii* parasites. This protein is a stage-specific protein, which presents low polymorphism between different *T. gondii* strains and elicits a strong specific antibody response [1, 2]. SAG1 is able to stimulate the production of IFN-γ of T cells in seropositive individuals [3]. For this reason, this antigen has been suggested as an excellent candidate for vaccine development in the prevention of toxoplasmosis.

In several studies, SAG1 has been used as a native-purified protein, recombinant protein or DNA expression vector in the development of vaccines against *T. gondii* [4, 5]. Likewise, various peptides of SAG1 have demonstrated to stimulate host humoral and cellular immunity and to provide partial protection against *T. gondii* [6]. The selection of proper epitopes becomes necessary to eliminate deleterious components and to retain the advantageous elements in the design of an efficient and safe vaccine [7, 8]. Therefore, many studies have identified different short peptides present in SAG1 that would have promising immunogenic properties and could be used in the development of acellular vaccines [9–12]. Wang et al. [11] showed that peptides of SAG1 containing B-cell epitopes are highly immunogenic. In addition, Godard et al. [9] observed that the C-terminus of SAG1 is the dominant antigenic and immunogenic region and, in particular, the SAG1_238–256_ peptide is an important T-cell epitope. Likewise, Siachoque et al. [10] scanned the B-cell epitopes present in SAG1 and revealed that they are primarily placed near the C-terminus. Furthermore, Cardona et al. [11] found that B-cell epitopes of SAG1 enclosed in the SAG1_301–320_ position show the highest reactivity against human sera from *T. gondii*-infected patients.

Another important aspect in the development of acellular vaccines is the selection of the appropriated adjuvant, since it plays an important role in the efficacy of the immunizations. It is generally accepted that a T helper 1 (Th1) response associated with IFN-γ producing cells, is the main mediator of the immunity against *T. gondii* infection [13, 14]. Therefore, Hsp90s and Hsp70s have been used as antigen/carriers in order to induce B- and T-cell mediated immune responses [15–19]. Our group showed that plant Hsp90s (pHsp90s) are B cell mitogens and that the presence of toll-like receptor 4 (TLR4) is necessary for a suitable response [18]. In addition, our results showed that pHsp90s could be incorporated as adjuvants in a vaccine formulation that needs a Th1-mediated response along with the stimulation of cytotoxic CD8^+^ cells to confer immunity [19].

Previously, we expressed SAG1 fused to *Leishmania infantum* 83-kDa heat-shock protein (LiHsp83-SAG1) to improve the stability of the recombinant protein when expressed in plants [20]. In addition, oral immunization with plant-made LiHsp83-SAG1 produced a significant reduction of the cyst load and this correlated with an increment of specific antibodies anti-rSAG1 [20]. A further advantage of employing Hsp90 as adjuvants/carriers is the possibility of using peptides containing B- and T-cell epitopes, instead of the full-length antigen or large polypeptides that may enclose toxic or unstable regions that can affect their correct expression in a heterologous system. While LiHsp83 is a pathogenic-associated adjuvant, pHsp90s offers the advantage of being a non-pathogenic adjuvant. Bearing this in mind, we characterized the role of pHsp90 in the modulation of the immune response. Thus, we evaluated the efficacy of *Nicotiana benthamiana* Hsp90.3 (NbHsp90.3) as a carrier/adjuvant of a T- and B-cell epitopes SAG1 peptide in a *T. gondii* infection model.

## Methods

### Plasmid construction

A peptide of *Toxoplasma gondii* SAG1 (SAG1_HC_) (from residue 221 to residue 319 of the protein) that encodes both T- and B-cell epitopes [10] was amplified by PCR using the plasmid pRSET-A-SAG1_77–322_ as a template [21]. The forward primer sequence was 5′-ggt acc ATA AAG TTC CTC AAG ACA AC-3′ and the reverse primer sequence was 5′-aag ctt CTA AAT GGA AAC GTG ACT GGC-3′ flanked by *Kpn*I and *Hind*III restriction sites (lowercase), respectively. The *N. benthamiana* Hsp90.3 full length sequence (NbHsp90.3) was amplified by PCR using the plasmid pRSET-A-NbHsp90.3 as a template [18]. The forward degenerated primer sequence was 5′-gga tcc ATG GCG GAS GCA GAR ACS TTT GCW TTY CAA GC-3′ and the reverse primer sequence was 5′-ctc gag GTC TAC TTC CTC CAT CTT TTC AGC ATC ATC AGC-3′ flanked by *Bam*HI and *Xho*I restriction sites (lowercase), respectively. The PCR products were first cloned separately into pGEM-T easy vectors (Promega, Fitchburg, WI, USA). After sequencing, the isolated segments were sequentially cloned into pRSET-A (Invitrogen, Carlsbad, CA, USA) to construct pRSET-A-NbHsp90.3-SAG1_HC_ expression vector.

### Expression and purification of recombinant proteins

The pRSETA-NbHsp90.3-SAG1_HC_ plasmid was transformed in *Escherichia coli* BL21 Star^™^(DE3) competent cells (Invitrogen). Bacteria were cultured to an optical density of 0.5 (absorbance at 600 nm) and then protein expression was induced with isopropyl-β-d-thiogalactoside (IPTG) to a final concentration of 1 mM for 4 h. Cells were harvested by centrifugation and stored at -20 °C until use.

Soluble NbHsp90.3-SAG1_HC_ protein was purified under non-denaturing conditions using a nitrilotriacetic acid-Ni^2+^ column (Qiagen, Germantown, MD, USA) [18]. Recombinant NbHsp90.3 protein was expressed in *E. coli* Rosetta strain (Invitrogen) and was purified as in Corigliano et al. [18]. Recombinant SAG1m (SAG1_77–322_) protein was expressed in *E. coli* and it was purified as in Laguía Becher et al. [21]. After purification, NbHsp90.3 (84.5-kDa), SAG1m (34-kDa) and NbHsp90.3-SAG1_HC_ (96-kDa) were passed through a polymyxin B agarose resin and then the LPS concentration was measured as previously described by Corigliano et al. [18].

### SDS-PAGE and Western blot analysis

Purified recombinant proteins were separated by SDS-PAGE (10%) using the Mini-Protean system III (Bio-Rad, Hercules, CA, USA) and stained with Coomassie Brilliant Blue. For Western blot analysis, recombinant proteins were transferred onto PVDF membranes (GE Healthcare, Chicago, IL, USA) using an Electro Transfer Unit (Bio-Rad). The membranes were incubated with either mouse anti-6xHIS monoclonal antibody (1:1000; Cell Signaling Technology Inc., Danvers, MA, USA), mouse polyclonal antibodies against rSAG1m (1:1000) or sera from NbHsp90.3-SAG1_HC_ immunized mice (1:100) as primary antibodies. Then, membranes were incubated with alkaline phosphatase conjugated goat anti-mouse IgG (1:5000, Sigma, St. Louis, MO, USA). The reaction was developed by the addition of nitroblue tetrazolium/5-bromo-4-chloro-3-indolyl phosphate (NBT/BCIP, Promega) substrate. PageRuler^™^ Prestained Protein Ladder (Fermentas, Waltham, MA, USA) was used as molecular marker.

### Preparation of the total lysate antigens (TLA) from *Toxoplasma gondii* tachyzoites

TLA were obtained from a suspension of *T. gondii* RH tachyzoites. RH tachyzoites were sonicated (10 s pulse ON with 30 s intervals OFF, for 1 min) using a 130 W ultrasonic processor (Vibra Cell^™^; Sonic, Newtown, CT, USA) and centrifuged at 8000× *g* for 5 min at 4 °C. Then, the supernatant was quantified by Bradford protein assay and stored at -80 °C until use.

### Mice and vaccination

C57BL/6 (H-2b) mice specific-pathogen-free were obtained from the central bioterium of Facultad de Ciencias Exactas y Naturales of Universidad de Buenos Aires. Six- to eight-week-old female mice were bred and housed following the institutional guidelines of the Universidad de General San Martín (C.I.C.U.A.E., IIB-UNSAM, 09/2016). Mice had access to food and water *ad libitum* and were maintained at 21–22 °C, 12:12 h light-dark photocycle. Five to eight mice per group were intraperitoneally immunized with equimolar amount of SAG1m (34-kDa, 2.5 μg), NbHsp90.3 (84.5-kDa, 7.5 μg) and NbHsp90.3-SAG1_HC_ fusion protein (96-kDa, 9 μg). Each group received 4 doses (every 15 days) of each protein or PBS control. One day before immunizing, mice were bled from the tail vein and sera were stored at -20 °C until analysis.

### Antibody titers and isotype determination

Anti-rSAG1m and anti-rNbHsp90.3 antibodies in the sera of vaccinated mice were determined by ELISA. Briefly, 96-well microtiter plates (Nunc MaxiSorp^™^ flat-bottom, Thermo Fisher, Waltham, MA, USA) were coated overnight at 4 °C with 5 µg/ml of rSAG1m or 50 µg/ml of TLA (SAG1_TLA_). Serial dilutions of mice sera were carried out to determine the total IgG (IgGt) titer. The cut-off value was defined as the media of pre-immune sera absorbance values plus three standard deviations. For isotype determination, mice sera were diluted 1:100. Either goat anti-mouse IgG-horseradish peroxidase conjugated (1:5000) (Sigma-Aldrich, St. Louis, MO, USA), rat anti-mouse IgG1, IgG2a or IgG2b horseradish peroxidase conjugated (1:10000) (Sigma-Aldrich) were used as secondary antibodies. Tetramethyl-benzidine substrate (TMB; Invitrogen) was added and plates were read at 655 nm with an automatic ELISA reader (Synergy H1; Bio-Tek, Winooski, VT, USA).

### Cytokine analysis

The cytokine production was evaluated in independent experiments, either 15 days after the last immunization (pre-challenge) or 30 days after challenging with 20 Me49 tissue cysts (post-challenge). Spleens from 5 immunized mice from each group were removed and the splenocytes (1.25 × 10^6^ cells/well) were stimulated with either 10 µg/ml of rSAG1m, rNbHsp90.3 or rGRA4. After 72 h, the production of IL-4, IL-10 and IFN-γ in harvested cell-free supernatants was assessed as previously described by Corigliano et al. [19].

### Challenge infection

Two weeks after the last immunization, mice were orally challenged with 20 tissue cysts of *T. gondii* Me49 strain as previously described [21]. Mice were observed daily for mortality determination. One month after the challenge, animals were sacrificed, and their brains were removed for tissue cyst quantification. Each brain was homogenized in 2 ml of PBS by eight passages through a 21-gage needle [22]. The mean number of cysts per brain was determined by observation with an optical microscope, by counting four samples of 20 μl aliquots of each homogenized brain [21].

### Statistical analysis

Statistical analysis was carried out with Prism 5.0 (GraphPad, San Diego, CA, USA) using one- and two-way analysis of variance (ANOVA). Statistical analysis was carried out using Tukey’s multiple comparisons test to compare means among groups. Values of *P* < 0.05 were considered significantly different.

## Results

### Expression of recombinant proteins

We expressed peptide of *Toxoplasma gondii* SAG1 from aa_221_ to aa_319_ (SAG1_HC_). This peptide encloses B- and T-cell epitopes [10]. The peptide was cloned in frame with *Nicotiana benthamiana* Hsp90.3 (NbHsp90.3) [18] and was expressed and purified from *E. coli*.

### Humoral immune response generated by NbHsp90.3-SAG1_HC_ fusion protein

Mice were intraperitoneal immunized with the NbHsp90.3-SAG1_HC_ fusion protein (NbHsp90.3-SAG1_HC_ group), SAG1m (SAG1_m_ group), NbHsp90.3 (NbHsp90.3 group) or PBS buffer 1× (PBS group). In order to administrate approximately equimolar quantities of recombinant proteins, the immunization doses for each antigen formulation were: SAG1m (34-kDa, 2.5 μg), NbHsp90.3 (84.5-kDa, 7.5 μg) and NbHsp90.3-SAG1_HC_ fusion protein (96-kDa, 9 μg) of PBS 1×.

**Fig. 1:**
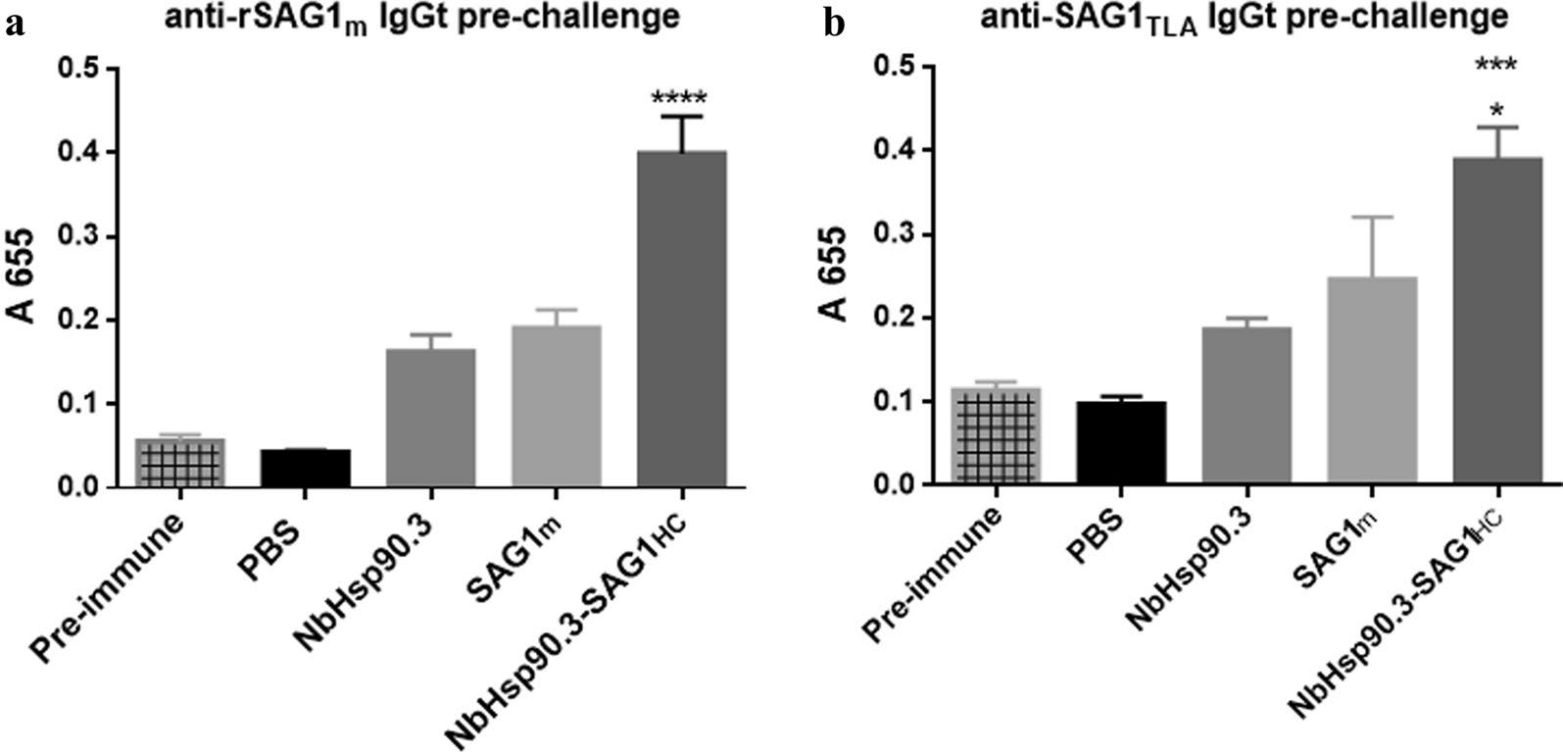
Levels of anti-SAG1 total IgG antibodies in the sera of C57BL/6 immunized mice. Two weeks after the last immunization, serum samples (8 mice per group) were collected to analyze IgGt by ELISA with rSAG1m (**a**) and SAG1_TLA_ (**b**) as the bound target. Sera were diluted 1:1000 or 1:100 for detecting rSAG1m or SAG1_TLA_, respectively. Each bar represents the group mean ± SEM. Results represent one of three similar experiments. Pre-immune sera were used as a negative control. **a** ****P < 0.0001, NbHsp90.3_HC_ *vs* PBS, NbHsp90.3 and SAG1m groups. **b** *P < 0.05, NbHsp90.3_HC_ *vs* SAG1m group; ***P < 0.001, NbHsp90.3_HC_ *vs* PBS and NbHsp90.3 groups. Statistical analysis was performed by one-way analysis of variance (ANOVA) using Tukey’s multiple comparisons test

The efficacy of vaccination in immunized mice was determined by serological analysis. First, we analyzed by Western blot whether the sera from mice immunized with the different formulations were able to recognize SAG1 antigen present in TLA (SAG1_TLA_). The sera pools from mice immunized with NbHsp90.3-SAG1_HC_ fusion protein or SAG1m were able to immunodetect SAG1_TLA_ (Additional file 1: Figure S1). Therefore, we used SAG1_TLA_ and recombinant SAG1m (rSAG1m) in all serological analysis. Production of IgG antibodies against rSAG1m and SAG1_TLA_ was evaluated by ELISA 60 days after the first immunization. The levels of anti-rSAG1m and anti-SAG1_TLA_ total IgG antibodies were strongly increased in NbHsp90.3-SAG1_HC_-immunized mice (Fig. 1). In fact, significant differences were observed between the NbHsp90.3-SAG1_HC_ group and control groups (SAG1m- NbHsp90.3- and PBS-immunized mice) (Fig. 1). Therefore, NbHsp90.3-SAG1_HC_ formulation elicits a strong humoral response in comparison with SAG1m group, suggesting that NbHsp90.3 works as an efficient adjuvant to stimulate B-cell epitopes when associated to the antigen of interest. In addition, no anti-rSAG1m or anti-SAG_TLA_ IgGt was detected in the PBS or NbHsp90.3 groups (Fig. 1).

**Fig. 2:**
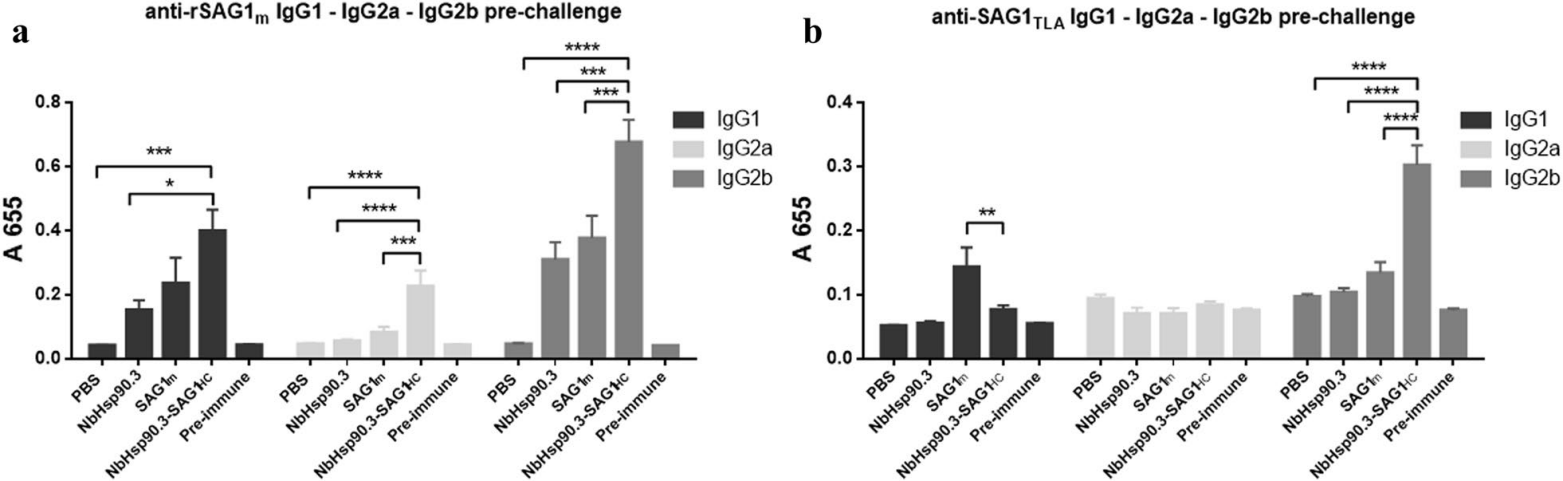
Analysis of IgG isotype antibodies against SAG1 in the sera of C57BL/6 immunized mice. Two weeks after the immunization schedule was completed, serum samples (8 mice per group) were collected and diluted 1:100 to analyze IgG1, IgG2a and IgG2b sub-classes with rSAG1m (**a**) or SAG1_TLA_ (**b**) as the bound target. Each bar represents the group mean ± SEM. Results represent one of three similar experiments. Pre-immune sera were used as a negative control. *P < 0.05; ***P < 0.001; ****P < 0.0001. Statistical analysis was performed by two-way analysis of variance (ANOVA) using Tukey’s multiple comparisons test

To investigate the isotype profile induced after immunization, we evaluated the presence of IgG1, IgG2a and IgG2b antibodies in serum samples on day 60, by using rSAG1m or SAG1_TLA_ as the bound targets (Fig. 2). All NbHsp90.3-SAG1_HC_-immunized mice produced IgG1 and IgG2a antibodies that were able to recognize rSAG1m but not SAG1_TLA_ (Fig. 2). In addition, NbHsp90.3-SAG1_HC_-immunized mice produced high levels of anti-SAG1_TLA_ IgG2b antibody isotype, whereas SAG1m-immunized mice showed a predominant specific IgG1 response against SAG1_TLA_. Furthermore, higher levels of anti-rSAG1m IgG2b antibodies were observed in NbHsp90.3 and SAG1m groups respect to PBS group, which were not observed when the SAG1_TLA_ was used as the bound target (Fig. 2). This could be due to the shared N-terminus sequences for both recombinant proteins.

### Cellular immune response generated by NbHsp90.3-SAG1_HC_ fusion protein

**Fig. 3:**
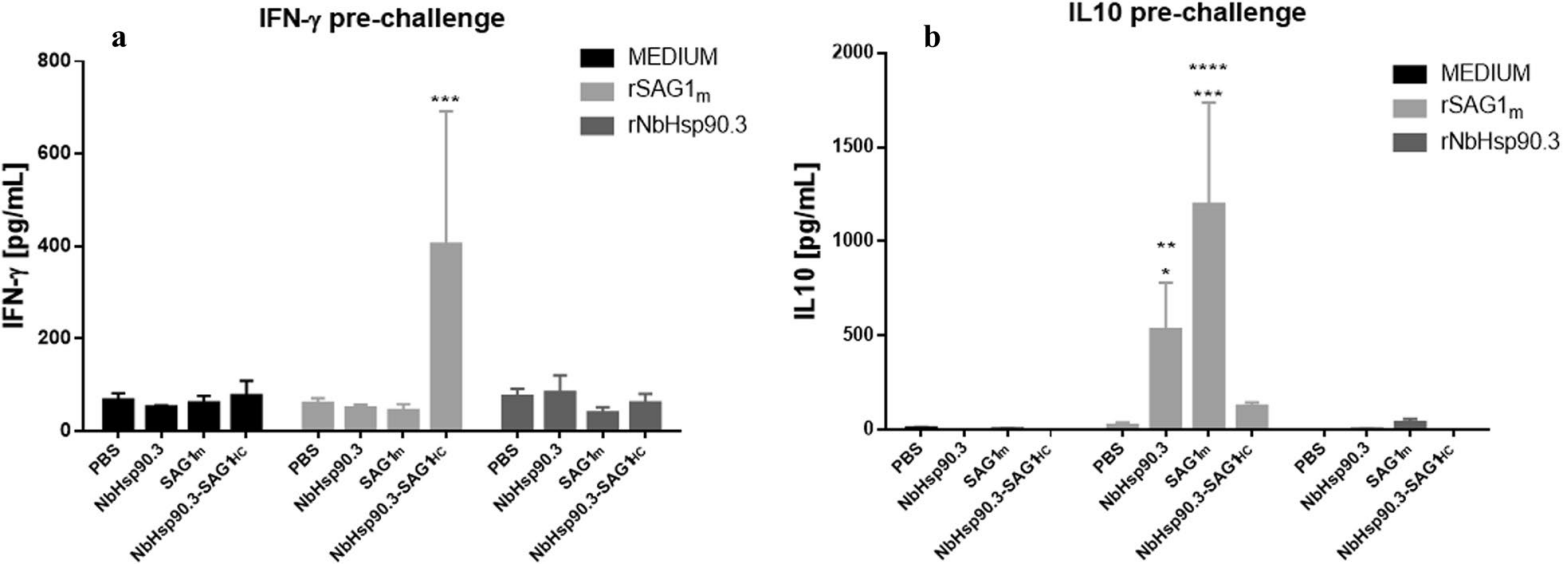
Cytokine production by spleen cell cultures from C57BL/6 immunized mice. Two weeks after last immunization, splenocytes from 5 mice per group were cultured, and the cytokine production in cell supernatants after rSAG1 stimulation was measured by ELISA. Values for IFN-γ (**a**) and IL-10 (**b**) at 72 h are expressed as mean ± SEM. Results represent one of two similar experiments. IFN-γ: ***P < 0.001 NbHsp90.3-SAG1_HC_ *vs* PBS, NbHsp90.3 and SAG1m groups. IL-10: *P < 0.05, NbHsp90.3 *vs* NbHsp90.3-SAG1_HC_ group; **P < 0.01, NbHsp90.3 *vs* PBS group; ***P < 0.001, SAG1m *vs* NbHsp90.3-SAG1_HC_ group; ****P < 0.0001 SAG1m *vs* PBS group. Statistical analysis was performed by two-way analysis of variance (ANOVA) using Tukey’s multiple comparisons test

To evaluate the cytokine response profile, splenocytes from vaccinated mice were stimulated with either rSAG1m or rNbHsp90.3 and the amounts of cytokine production (IFN-γ, IL-4 and IL-10) in different groups were measured. As shown in Fig. 3a, NbHsp90.3-SAG1_HC_-immunized mice generated a significantly higher level of IFN-γ compared with control groups (SAG1m-, NbHsp90.3- and PBS-immunized mice). Meanwhile, SAG1m and NHsp90.3 groups produced a significantly higher level of IL-10 compared with NHsp90.3-SAG1_HC_-immunized mice (Fig. 3b). IL-4 was not detected in any group (data not shown).

### Parasite load and humoral and cellular response after *T. gondii* infection

**Fig. 4:**
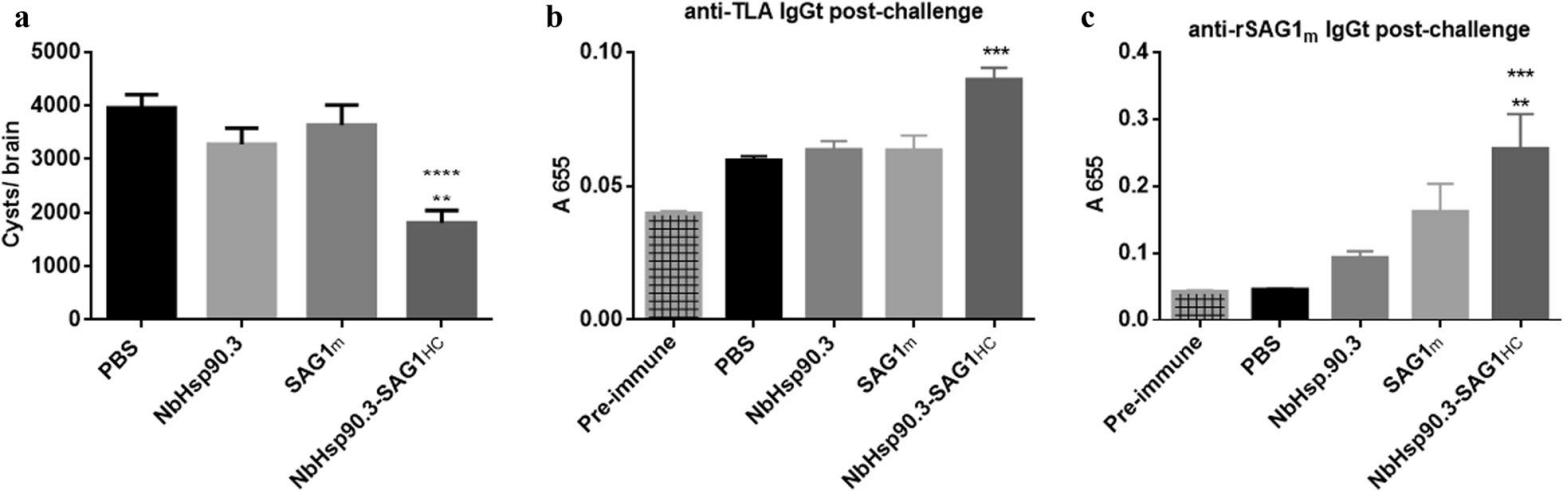
Post-challenge analysis in C57BL/6 immunized mice after *T. gondii* infection. **a** Protection against chronic *T. gondii* infection in C57BL/6 immunized mice. Two weeks after the last immunization, mice were orally challenged with 20 tissue cysts of ME49 *T. gondii* strain (sub-lethal dose). Thirty days after the infection, mice were sacrificed, and their brains were removed for cyst load determination. Each bar represents the group mean ± SEM. Results represent one of three similar experiments. **P < 0.01, NbHsp90.3-SAG1_HC_ *vs* NbHsp90.3 group; ****P < 0.0001, NbHsp90.3-SAG1_HC_ *vs* PBS and SAG1m groups. **b**, **c** Antigen-specific antibodies in sera from C57BL/6 immunized mice with chronic *T. gondii* infection. Thirty days after mice were orally challenged with 20 tissue cysts of ME49 *T. gondii* strain (sub-lethal dose), serum samples (8 mice per group) were obtained and IgGt was determined by ELISA with TLA (**b**) or rSAG1m (**c**) as the bound targets (sera were diluted 1:16,000 and 1:1000, respectively). Each bar represents the group mean ± SEM. Results represent one of two similar experiments. **b** ***P < 0.001, NbHsp90.3-SAG1_HC_ *vs* PBS, NbHsp90.3 and SAG1m groups. **c** **P < 0.01, NbHsp90.3-SAG1_HC_ *vs* SAG1m group; ***P < 0.001, NbHsp90.3-SAG1_HC_ *vs* PBS and NbHsp90.3 groups. Pre-immune sera were used as a negative control. Statistical analysis was performed by one-way analysis of variance (ANOVA) using Tukey’s multiple comparisons test

**Fig. 5:**
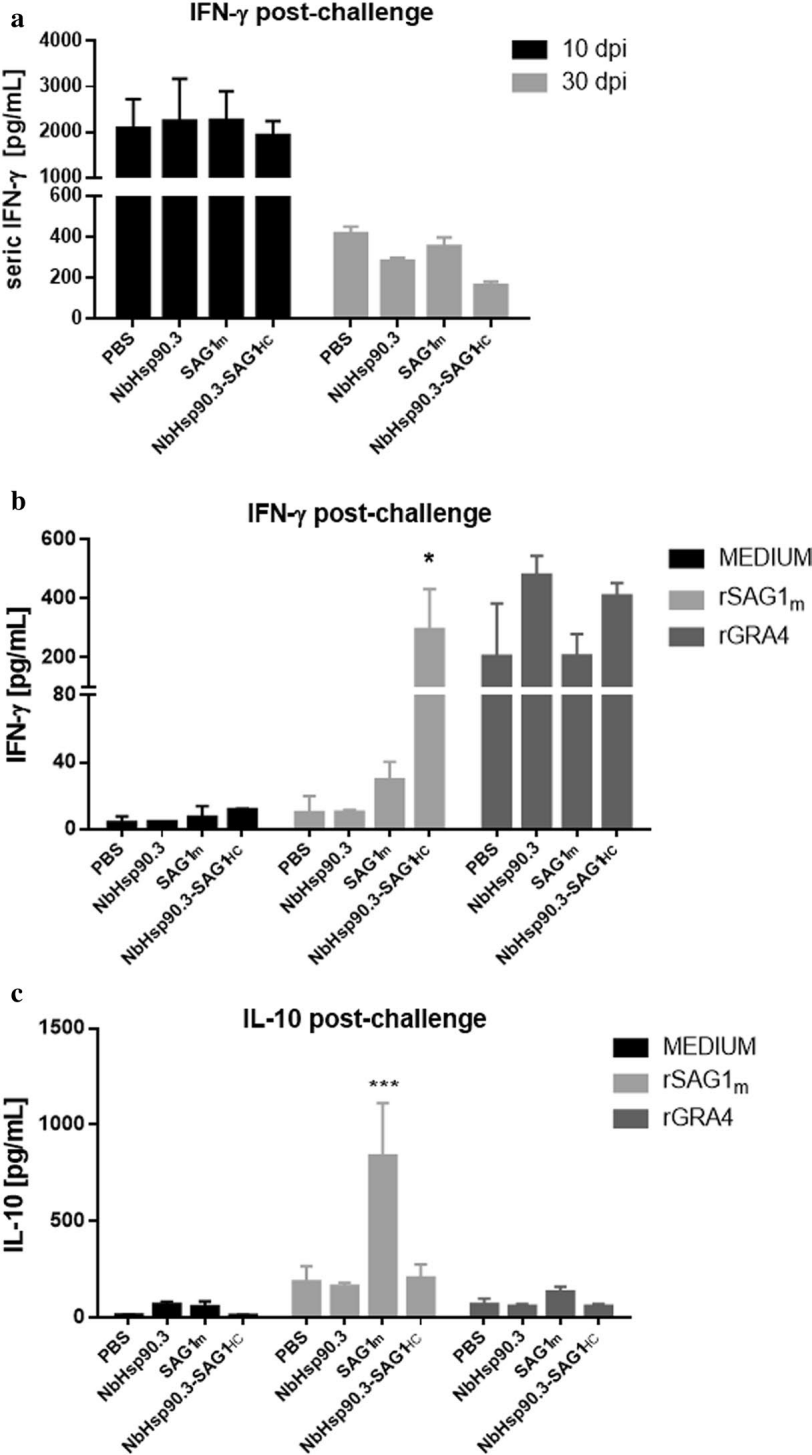
Cytokine detection in spleen cell cultures and sera from C57BL/6 immunized mice after *T. gondii* infection. **a** Values of IFN-γ in serum samples (5 mice per group) at 10 days (acute infection) and 30 days (chronic infection) after mice were orally challenged with 20 tissue cysts of ME49 *T. gondii* strain (sub-lethal dose). Sera were diluted 1:10 and IFN-γ was measured by ELISA. Values of IFN-γ (**b**) and IL-10 (**c**) in supernatants of spleen cell cultures in the chronic infection. Splenocytes from 5 mice per group were cultured and stimulated with either rSAG1 or rGRA4. Cytokine was measured in cell supernatants after 72 h of culture by ELISA. Each bar represents the group mean ± SEM. Results represent one of two similar experiments. IFN-γ: *P < 0.05, NbHsp90.3-SAG1_HC_ *vs* PBS, NbHsp90.3 and SAG1m groups. IL-10: ***P < 0.001, NbHsp90.3-SAG1_HC_ *vs* PBS, NbHsp90.3 and NbHsp90.3-SAG1_HC_ groups. Statistical analysis was performed by two-way analysis of variance (ANOVA) using Tukey’s multiple comparisons test

To assess the effect on parasite load against *T. gondii* infection, two weeks after the vaccination schedule was completed, immunized mice were orally challenged and the number of *T. gondii* brain cysts was analyzed one month later. All mice survived for 30 days. The behavior of NbHsp90.3-SAG1_HC_-immunized mice was apparently normal, while SAG1m-, NbHsp90.3- and PBS-immunized mice exhibited lack of movement and a general aspect indicating illness. While the brain cyst loads in SAG1- and NbHsp90.3-immunized mice were not significantly lower compared to PBS-immunized mice, the NbHsp90.3-SAG1_HC_-immunized mice presented a significant reduction in the cyst load (56%) when compared to control groups (SAG1m-, NbHsp90.3- and PBS-immunized mice) (Fig. 4a). In addition, we evaluated the humoral immune response in the chronic phase of *T. gondii* infection (Fig. 4b, c). Thirty days after the challenge, the levels of anti-TLA IgGt were significantly increased in all groups (Fig. 4b), especially in the NbHsp90.3-SAG1_HC_-immunized mice (Fig. 4b). By contrast, high levels of anti-rSAG1m IgGt antibodies were detected in NbHsp90.3-SAG1_HC_- and SAG1m-immunized mice, while no response was observed in NbHsp90.3 and PBS groups (Fig. 4c). Furthermore, we evaluated the cytokine profile in sera from immunized and challenged mice during the acute and chronic phases of *T. gondii* infection (10 and 30 days after the challenge, respectively). The levels of IFN-γ were induced in the acute phase but they were significantly diminished in the sera from all mice with chronic infection. Actually, no significant differences were observed among groups (Fig. 5a). On the other hand, IL-10 and IL-4 were undetectable in sera from all mice with acute and chronic infection (data not shown). In addition, the cytokine profile was analyzed in supernatant of spleen cell cultures. IL-10, IL-4 and IFN-γ production was measured in the supernatants from spleen cells culture of immunized mice in the chronic phase of *T. gondii* infection. Splenocytes were stimulated *in vitro* with rSAG1m and recombinant GRA4 (rGRA4) (Fig. 5b). The stimulation with rGRA4 was carried out in order to distinguish the cellular response induced by the *T. gondii* challenge from the cellular response induced by the vaccination and the challenge. As expected, after the challenge, IFN-γ levels were strongly induced in all groups when spleen cells were stimulated with rGRA4 (Fig. 5b), while IL-10 was not secreted (Fig. 5c). On the other hand, the spleen cells from SAG1m- and NbHsp90.3-SAG1_HC_-immunized mice stimulated with rSAG1m showed different cytokine profile production (Fig. 5b, c). While, the levels of IFN-γ were significantly induced in the NbHsp90.3-SAG1_HC_-immunized mice (Fig. 5b), IL-10 was only secreted by splenocytes from SAG1m-immunized mice (Fig. 5c). Notably, splenocytes from NbHsp90.3- and PBS-immunized mice stimulated with rSAG1m did not show an increase of any of the analyzed cytokines. Finally, IL-4 was not secreted by spleen cells of any of the groups stimulated with either rGRA4 or rSAG1m (data not shown).

## Discussion

Several Hsp90s from different organisms, in particular Gp96, are being studied as promising adjuvants to enhance innate and adaptive immune responses, especially as potent T cell activators for designing vaccines against infectious diseases [19, 23–28]. According to these properties, Hsp90s have been explored as adjuvants for enhancing vaccine potency [28–30]. The most useful, simple and feasible strategy employed is to covalently link the desired antigen to Hsp90. In previous studies, it was demonstrated that immunization with Hsp90 from *Leishmania infantum* (LiHsp83) fused to a reporter antigen (MBP-LiHsp83) or a *T. gondii* Rop2 antigen (Rop2-LiHsp83), generated mainly IgG2a isotype antibodies inferring the involvement of an immune response mediated by Th1 cells [23, 24, 31]. Like other Hsp90s, the Hsp90.3 isoform from *Nicotiana benthamiana* (NbHsp90.3) is also considered a promising adjuvant with the advantage that NbHsp90.3 comes from a non-pathogenic source and it can be used for any pathogen-associated antigens, whereas LiHsp83 could interfere with antigens from other pathogens such as trypanosomatids. In agreement with this, we previously demonstrated that BALB/c mice immunized with MBP-NbHsp90.3 were able to elicit a Th1 immune response against MBP [19]. Since analysis with generic antigens will not necessarily reproduce the immune response against microbial- or parasitic-derived antigens, we aimed to evaluate the potential of NbHsp90.3 as a carrier/adjuvant of a peptide from *T. gondii* SAG1 protein.

SAG1 is considered an important antigen for the development of effective diagnostic tests or subunit vaccines [6]. Significant progress has been made towards characterization of its immunogenicity and a significant amount of information is available to date on the different antigenic regions related to T- or B-cell responses [10, 12, 32–34]. In the present study, we analyzed both humoral and cellular immune responses induced by NbHsp90.3 covalently linked to SAG1_HC_ (a peptide that contains a T- and B-cell epitopes) [10]. NbHsp90.3, SAG1m or PBS were defined as control groups and the experiments were carried out in the murine model.

Humoral response analysis showed that NbHsp90.3-SAG1_HC_-immunized mice induced a high production of specific-antibodies that were able to recognize rSAG1m and also SAG1_TLA_. Sera from NbHsp90.3-SAG1_HC_-immunized mice showed a significant increase of anti-rSAG1m IgG2a and IgG2b antibody isotypes and only anti-SAG1_TLA_ IgG2b antibodies. This finding suggests that the B-cell epitope present in the SAG1_HC_ peptide would generate antibodies which are able to distinguish rSAG1m from SAG1_TLA_. Another interesting result is that the SAG1-immunized mice showed a significative increase of the anti-SAG1_TLA_ IgG1 levels, suggesting that humoral immune response induced by NbHsp90.3-SAG1_HC_ formulation is addressed towards a Th1 profile when the antigenic peptide is carried by NbHsp90.3. These outcomes are consistent with those found by Corigliano et al. [19]. However, it must be noted that while in the present study SAG1_HC_ is fused to the C-terminus of NbHsp90.3, the carried protein was fused to the N-terminus in Corigliano et al. [19]. Therefore, the adjuvant properties present in NbHsp90.3 will be maintained independently where the carrier protein is situated. It is also worthy of mention that in order to trigger a specific humoral immune response against the carried antigen, it is not necessary to fuse the complete protein of interest to NbHsp90.3, but only those epitopes with antigenic capacity.

As cytokines play an important role in the protection against *T. gondii* infection, we assessed their production. Our previous studies showed that NbHsp90.3 elicited a high production of IFN-γ when used as adjuvant [19]. It is important to note that IFN-γ is a key mediator in the mechanisms of *T. gondii* resistance and plays an important role in protecting hosts during both acute and chronic phases of toxoplasmosis [35–37]. In this study, we observed that splenocytes from NbHsp90.3-SAG1_HC_-immunized mice produced the highest amounts of IFN-γ when stimulated with rSAG1m. These results confirm that the presence of the selected T- and B-cell epitopes of SAG1 is enough to elicit a Th1 immune response when they are fused to NbHsp90.3. On the other hand, it is generally assumed that IL-10 production correlates with a Th2 T-cell response [38]. In our study, high levels of IL-10 were elicited by spleen cells from mice of control groups (SAG1m and NbHsp90.3 groups). Actually, the high levels of IL-10 could explain the absence of IFN-γ in the SAG1m- and NbHsp90.30-immunized mice. According to these results, the immunization with SAG1m alone would induce a Th2 immune response with a production of IgG1 antibodies and IL-10 secretion. In fact, several lineal and conformational B-cell epitopes have been identified in SAG1 protein, most of them localized in the N-terminus region [39]; thus, it is expected that immunization with SAG1m alone would induce a Th2 immune response.

As the main goal of vaccination is the stimulation of a protective immune response against a defined pathogen, immunized mice were infected with *T. gondii*. In order to assess the protective immunity against *T. gondii* infection, the humoral and cellular immune responses were analyzed after the challenge. Interestingly, while antibodies from NbHsp90.3-SAG1_HC_-immunized mice were able to respond to rSAG1m and SAG1_TLA_, the antibodies generated after the infection were unable to react with rSAG1m, but they did with the TLA extract. This could be explained by the posttranslational modification that takes place to the native SAG1 into the parasite [32] which could contribute in modifying the conformational profile of epitopes of both proteins. Similar results were observed by Sánchez et al. [40]. The authors showed that sera from mice infected with *T. gondii* were unable to recognize the recombinant TgPI-1 protein [40]. Taken together, these results suggest that rSAG1m y SAG1_TLA_ carried dissimilar epitopes that make it possible to differentiate between vaccinated and infected mice.

On the other hand, the analysis of cytokines after the challenge in the chronic phase of *T. gondii* infection showed that rGRA4-stimulated spleen cells enhanced a high production of IFN-γ without secretion of either IL-10 or IL-4, indicating the *T. gondii* infection elicited a strong adaptative Th1 T cell immune response in mice from all groups. Only rSAG1m-stimulated spleen cells from NbHsp90.3-SAG1_HC_ group also showed high production of IFN-γ in agreement with what was observed before the challenge. In addition, SAG1m group triggered a Th2 adaptative immune response against rSAG1m indicating that *T. gondii* infection did not modify the cytokine profile against the SAG1m antigen elicited by the immunization. Taken together these results indicate that the NbHsp90.3 protein not only enhances cell memory response against the carried antigen but also modulates the response towards a Th1 profile in the immunized mice, which could be responsible for the conferred immune protection against *T. gondii* infection. More importantly, NbHsp90.3-SAG1_HC_-immunized mice showed a significant reduction in the parasitic load (56%) with respect to the other groups concurrently in agreement with the cellular and humoral response observed in this group. Therefore, the capability of NbHsp90.3-adjuvanted vaccines to induce memory T cells can respond more rapidly to challenge and provide a long-term protective anti-*T. gondii* immunity. Although the percentage of cyst load reduction is comparable to others immunized with recombinant proteins [40–44], we emphasize that in the NbHsp90.3-SAG1_HC_ group no exogenous adjuvant was added. In summary, these results indicate that the cellular and humoral immune responses induced by NbHsp90.3-SAG1_HC_ formulation are addressed towards a Th1 immune profile with a production of IFN-γ, IgG2a and IgG2b antibodies against the carried antigen. We demonstrated the ability of NbHsp90.3 as an excellent adjuvant that modulates the immune response against *T. gondii* antigens in an appropriate manner.

## Conclusions

In the present study we validated the use of NbHsp90.3 as an adjuvant in a murine model. We developed and validated a vaccine formulation which, to our knowledge, for the first time includes the NbHsp90.3 protein covalently fused to a peptide from *T. gondii* SAG1 protein that contains T- and B-cell epitopes. We also demonstrated that NbHsp90.3 enhances a humoral and cell- mediated immune response along with Th1 cytokine production. After the challenge, mice vaccinated with NbHsp90.3-SAG1_HC_ exhibited a partial protection against the *T. gondii* infection which correlated with the induction of the memory immune response. In conclusion, NbHsp90.3 is an attractive adjuvant to be incorporated in vaccine formulations that require the generation of a Th1 profile along with a T-cell mediated response to confer immunity.

## Additional file

**Additional file 1: Figure S1.** Immunoblot profiles of extract from tachyzoite lysate antigens (TLA) probed with sera (1:100) from PBS-immunized mice (Lane 1), NbHsp90-immunized mice (Lane 2), SAG1m-immunized mice (Lane 3), NbHsp90.3-SAG1_HC_-immunized mice (Lane 4) and with mouse anti-rSAG1m polyclonal antibody (1:1000) (C+). The arrow indicates the band that reacted with SAG1m- and NbHsp90.3-SAG1_HC_-positive sera and anti-rSAG1m polyclonal antibody. M: molecular weight marker (Fermentas).

## Abbreviations

Hsp90: 90-kDa heat-shock protein
NbHsp90.3: Hsp90.3 isoform from *Nicotiana benthamiana*
rSAG1m: mature SAG1 protein from *Toxoplasma gondii*
SAG1_HC_: peptide of SAG1 protein from *T. gondii*
NbHsp90.3-SAG1_HC_: fusion protein containing NbHsp90.3 and SAG1_HC_
TLA: total lysate antigen extract from *T. gondii* tachyzoites
SAG1_TLA_: native SAG1 present in TLA
