# Supplementary figures and images for: Plant Hsp90 is a novel adjuvant that elicits a strong humoral and cellular immune response against B- and T-cell epitopes of a Toxoplasma gondii SAG1 peptide
Source: Parasit Vectors. 2019 Mar 25;12:140. doi: 10.1186/s13071-019-3362-6 (PMC6434815; doi:10.1186/s13071-019-3362-6)

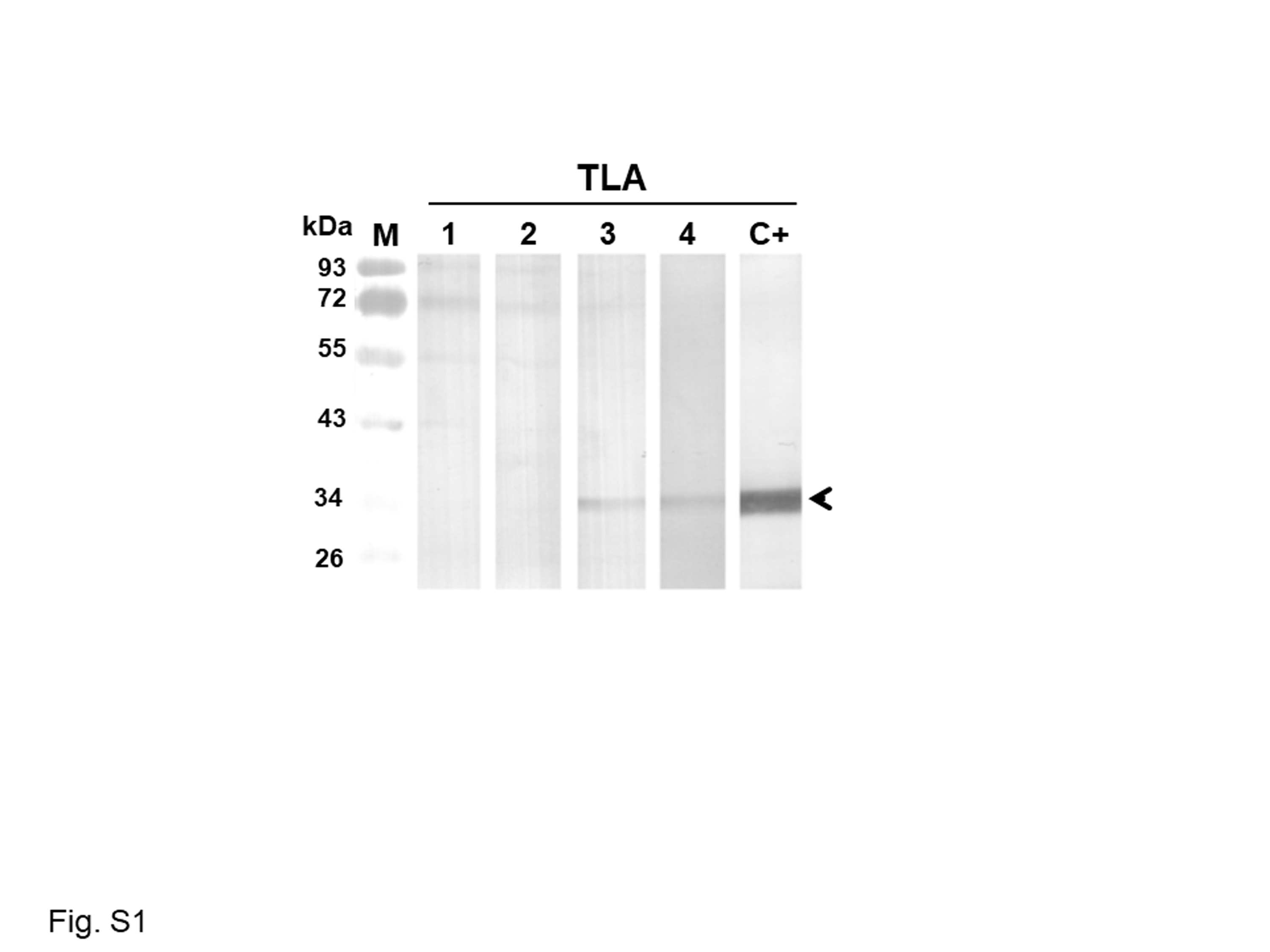

Supplement: Supplementary file 1 — Additional file 1: Figure S1. Immunoblot profiles of extract from tachyzoite lysate antigens (TLA) probed with sera (1:100) from PBS-immunized mice (Lane 1), NbHsp90-immunized mice (Lane 2), SAG1m-immunized mice (Lane 3), NbHsp90.3-SAG1HC-immunized mice (Lane 4) and with mouse anti-rSAG1m polyclonal antibody (1:1000) (C+). The arrow indicates the band that reacted with SAG1m- and NbHsp90.3-SAG1HC-positive sera and anti-rSAG1m polyclonal antibody. M: molecular weight marker (Fermentas). [file 13071_2019_3362_MOESM1_ESM.tif]
